# Immune reactivity to *Trypanosoma cruzi* chimeric proteins for Chagas disease diagnosis in immigrants living in a non-endemic setting

**DOI:** 10.1186/s12879-019-3872-z

**Published:** 2019-03-12

**Authors:** Eva Dopico, Rodrigo Pimenta Del-Rei, Bertha Espinoza, Itziar Ubillos, Nilson Ivo Tonin Zanchin, Elena Sulleiro, Zaira Moure, Paola Alejandra Fiorani Celedon, Wayner Vieira Souza, Edimilson Domingos da Silva, Yara Miranda Gomes, Fred Luciano Neves Santos

**Affiliations:** 1 Laboratori Clínic Territorial Metropolitana Sud, Catalan Institute of Health, Barcelona, Catalonia Spain; 2 Faculty of Technology and Sciences of Bahia, Salvador, Bahia Brazil; 3 Instituto de Investigaciones Biomédicas, Departamento de Inmunología, Universidad Nacional Autónoma de México, Ciudad de México, Mexico; 4 Carlos Chagas Institute, Oswaldo Cruz Foundation, Curitiba, Paraná Brazil; 5 Microbiology Department, Vall d’Hebron University Hospital, PROSICS, Barcelona, Catalonia Spain; 6 Molecular Biology Institute of Paraná, Curitiba, Paraná Brazil; 7 Aggeu Magalhães Institute, Oswaldo Cruz Foundation, Recife, Pernambuco Brazil; 8 Immunobiological Technology Institute, Oswaldo Cruz Foundation, Rio de Janeiro, Brazil; 9 Gonçalo Moniz Institute, Oswaldo Cruz Foundation, Salvador, Bahia Brazil

**Keywords:** Chagas disease, *Trypanosoma cruzi*, Chimeric antigens, Immunoassay, Accuracy

## Abstract

**Background:**

Chronic Chagas Disease (CD) diagnosis is based on serological methods employing crude, semipurified or recombinant antigens, which may result in low sensitivity or cross-reactivity. To reduce these restrictions, we developed a strategy involving use of molecules containing repetitive fragments of *Trypanosoma cruzi* conserved proteins. Diagnostic performance of IBMP-8.1 and IBMP-8.4 chimeric antigens (Molecular Biology Institute of Paraná - IBMP in Portuguese acronym) was assessed to diagnose *T. cruzi*-infected and non-infected immigrants living in Barcelona (Spain), a non-endemic setting for Chagas disease.

**Methods:**

Reactivity of IBMP-8.1 and IBMP-8.4 was assessed using an in-house automated ELISA with 347 positive and 331 negative individuals to Chagas disease. Antigenic cross-reactivity was measured with sera samples from pregnant women with *Toxoplasma gondii* (*n* = 98) and Zika virus (*n* = 75) antibodies.

**Results:**

The area under the curve values was 1 and 0.99 for the IBMP-8.1 and IBMP-8.4 proteins, respectively, demonstrating excellent diagnostic accuracy. The reactivity index was higher for IBMP-8.1 than IBMP-8.4 in positive samples and no significant difference in reactivity index was observed in negative samples. Sensitivity ranged from 99.4% for IBMP-8.1 to 99.1% for IBMP-8.4 and was not statistically different. Specificity for IBMP-8.1 reached 100 and 99.7% for IBMP-8.4, both nearly 100% accurate. No antigenic cross-reactivity was observed and reactivity index was similar to that for negative Chagas disease individuals.

**Conclusions:**

Our results showed an outstanding performance of IBMP-8.1 and IBMP-8.4 chimeric antigens by ELISA and suggest both chimeric antigens could also be used for Chagas disease diagnosis in immigrants living in non-endemic settings.

## Background

Chagas disease (CD) is a life-threatening infection caused by hemoflagellate protozoa *Trypanosoma cruzi*, generating an estimated of 14,000 deaths every year [1] and morbidity in 5.7 to 9.4 million people in the continental Western Hemisphere [2, 3]. The epidemiological pattern of CD has undergone substantial changes in last decades as a consequence of control campaigns in endemic countries, which have reduced vectorial and transfusional transmission [4]. Increasing international migration flows and more affordable traveling conditions from Latin America to non-endemic areas have contributed to epidemiology changes [5, 6]. CD is no longer limited exclusively to the impoverished rural regions of Latin America; it is transformed into a global health concern affecting people worldwide in both endemic and non-endemic countries and placing 100 million people at risk for acquiring the infection [7].

In Spain, there are more than 6 million immigrants, and more than 2 million (38%) are coming from CD endemic Latin America countries [8], posing CD as a public health challenge [9]. Indeed, Ecuador, Bolivia, and Argentina are the predominant areas of origin [7]. The diversity of the geographic areas leads to another challenge: the need for an accurate diagnostic test capable of identifying individuals infected with different *T. cruzi* strains. The high genetic variability of *T. cruzi* can be responsible for false negative results [10]. These false negative results could be avoided by using synthetic chimeric antigens with repetitive fragments of antigenic *T. cruzi* proteins for the detection of specific antibodies [11–14].

We performed ELISA [15, 16] and liquid microarray [17] to assess the potential diagnostic of four chimeric proteins, IBMP-8.1, IBMP-8.2, IBMP-8.3, and IBMP-8.4, to identify *T. cruzi*-infected individuals from several Brazilian endemic (Bahia, Goiás, Minas Gerais, and Pernambuco states; Brazil) and non-endemic settings (Paraná state; Brazil). These chimeric antigens were composed of immunodominant and conserved sequences, as described previously [15]. We obtained high-performance values and low cross-reactivity to *Leishmania* spp., a pathogen showing relatively high antigenic similarity to *T. cruzi* [15, 17]. Imprecision analyses showed that IBMP chimeric antigens are highly reproducible and IBMP-8.1 and IBMP-8.4 presented the highest performance values among the evaluated antigens. In this study, we endeavored to conduct an evaluation of the diagnostic performance of IBMP-8.1 and IBMP-8.4 chimeric antigens employing ELISA to diagnose *T. cruzi*-infected and non-infected immigrants living in Barcelona (Spain), a non-endemic setting for the CD.

## Methods

We used the methodology previously described by Santos et al. [18] and Brito et al. [19].

### Study samples

**Fig. 1:**
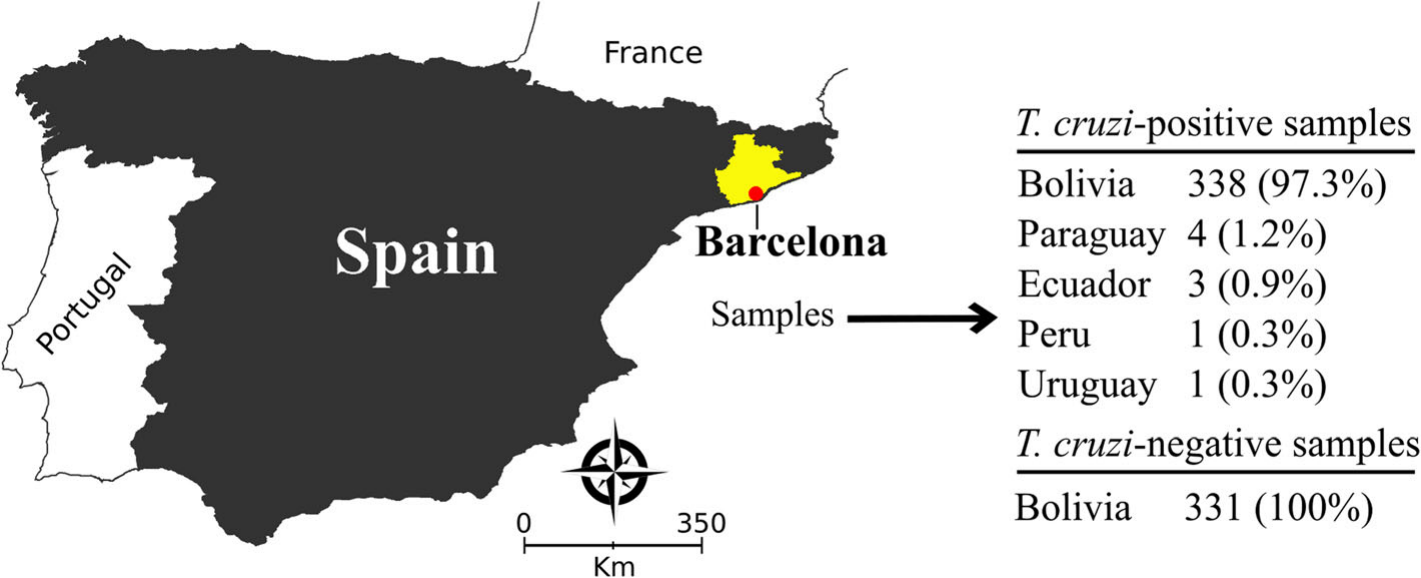
*T. cruzi*-positive and negative samples selected from Latin American immigrants living in Barcelona-Spain

We employed anonymized human sera from individuals diagnosed at the Laboratory Clínic l’Hospitalet-Laboratori Clínic Territorial Metropolitana Sur, Catalan Institute of Health (Barcelona-Spain). The minimum sample with a 95% confidence interval, an absolute expected error of 1.1% and sensitivity of 99% was 315 sera from non-infected and 315 from *T. cruzi*-infected individuals. We included sera from 331 non-infected and 347 *T. cruzi*-infected Latin American individuals living in Barcelona (Fig. 1). The sample selection was based on non-reactivity and reactivity by two serological assays: ORTHO® *T. cruzi* ELISA Test System (Ortho Clinical Diagnostics Inc., Raritan, USA), which employs *T. cruzi* whole cell lysate antigen; and Bioelisa CHAGAS (Biokit S.A., Barcelona, Spain) or BIO-FLASH® Chagas (automated chemiluminescent assay; Biokit S.A., Barcelona, Spain), the two latter composed by recombinant *T. cruzi* antigens. Samples with repeatedly discrepant results between both tests or inconclusive in one of them (or in gray zone) were defined as serodiscordant. Each sample was given an identifier code in the laboratory to ensure a blinded analysis. Antigenic cross-reactivity was assessed with sera samples from Latin American pregnant women with T*oxoplasma gondii* (*n* = 98) and Zika virus (ZIKV) antibodies (*n* = 75). Pregnant women sera samples were employed in this study due to the availability of the biological material in Laboratory Clínic l’Hospitalet-Laboratori Clínic Territorial Metropolitana Sur serum bank. Study participants were mostly from Bolivia (97.3%), but there were individuals from other CD endemic countries (Fig. 1).

### Recombinant antigens

Proteins were expressed in *Escherichia coli* Bl21 star DE3 and purified from the soluble fraction of the total extract of bacterial lysate. Both IBMP-8.1 and IBMP-8.4 antigens were purified by IMAC resin first, and the best fractions dialyzed for buffer exchange and salt reduction before following a second liquid chromatography step. The second purification was conducted on ionic exchange and heparin columns, respectively. Plasmidial construct has already been described in Santos et al. [18].

### Immunoassays

Assays were conducted according to previous reports [14, 16]. Briefly, polystyrene “Maxisorp” 96-well microplates (Nunc, Roskilde, Denmark) were coated with 25.0 ng of IBMP-8.1 and IBMP-8.4 per well diluted in coating buffer (0.05 M carbonate-bicarbonate, pH 9.6). Microplates were blocked with Well Champion reagent (Kem-En-Tec, Taastrup, Denmark) according to the manufacturer’s instructions. Serum samples were diluted in 0.05 M phosphate-buffered saline (pH 7.2)-0.5% Tween 20 (PBS-T), and 100 μl was added to each well. After 60 min of incubation at 37 °C, microplates were washed in PBS-T to remove unbound antibodies. HRP conjugated goat anti-human IgG (Bio-Manguinhos, FIOCRUZ, Rio de Janeiro, Brazil) was diluted 1:40,000 in PBS-T, and 100 μl were then added to each well, and the microplates were incubated for 30 min at 37 °C. Wells were washed five times and the immune complexes were revealed by the addition of 100 μl TBM substrate (tetramethyl-benzidine; Kem-En-Tec, Taastrup, Denmark). After a new cycle of incubation (10 min at RT in the dark), the reaction was stopped by adding 50 μl 5 N H_2_SO_4_, and the absorbance was measured at 450 nm. The protocols were automatized and the runs carried out in an automated microplate immunoanalyser (BEST 2000®, Biokit, Werfen Group Barcelona, Spain). The blank readings (buffer dilution) was subtracted from all other values.

### Statistical analysis

**Fig. 2:**
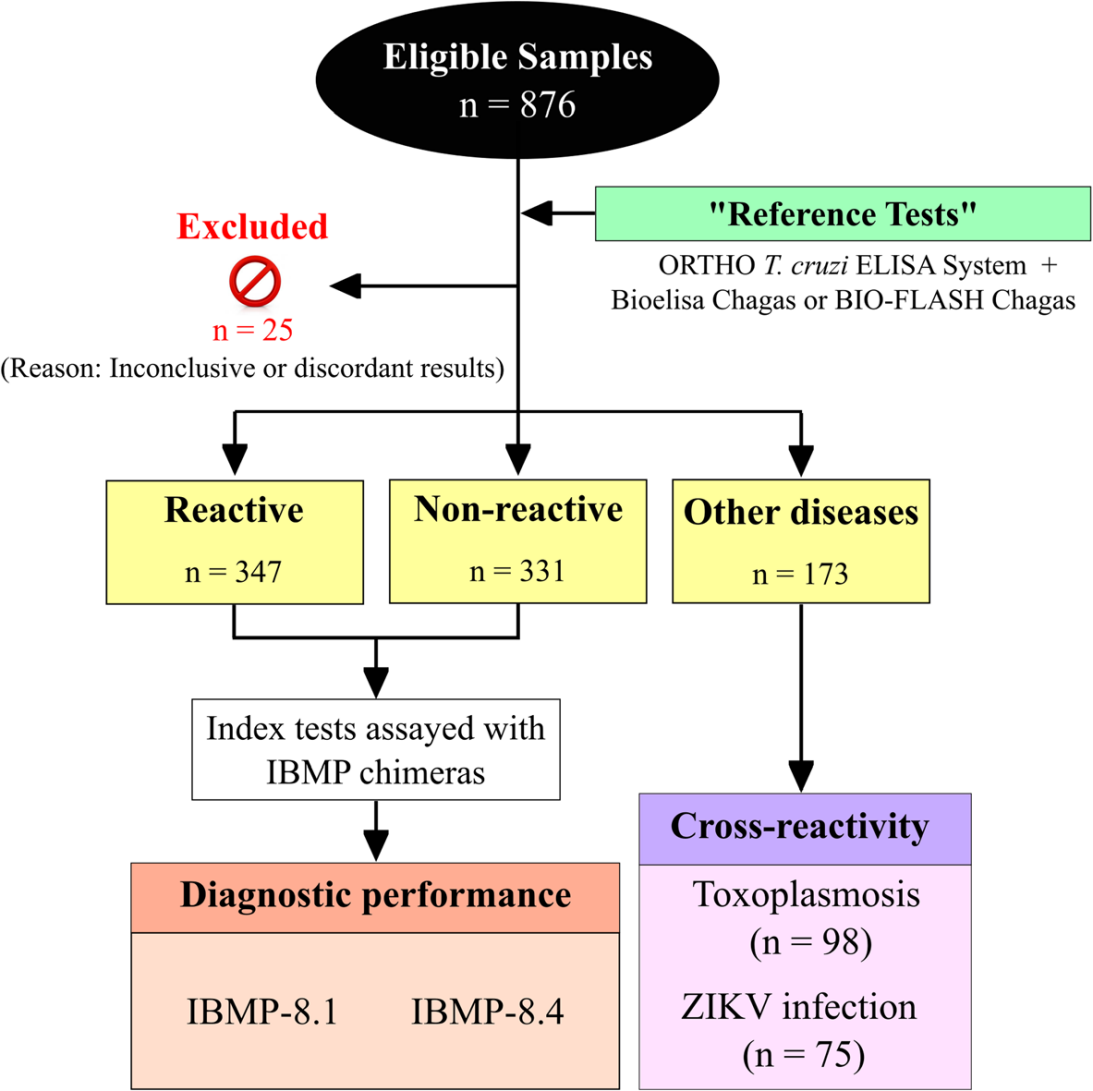
Study workflow for testing IBMP *T. cruzi* chimeras. RI, reactivity index; ZIKV, Zika virus

Data were coded and entered using computer graphic software (GraphPad Software Inc., La Jolla, CA, USA). Descriptive data were presented in the form of geometric means ± standard deviation. Shapiro-Wilk test, followed by Student’s t-test, was used to test data normality. When assumed homogeneity was not confirmed, Wilcoxon’s signed rank test was adopted. Cut-off values were determined under the receiver operating characteristic curve (ROC) analyzing the whole serum panel. For data normalization, all results were expressed by plotting values in an indexed format, calculated as the ratio between a given sample’s optical density (OD) and the cut-off OD values respective to each assay. Under this index, referred to as a reactivity index (RI), all results ≥1.00 were considered positive. When a sample’s RI value was 1.0 ± 10%, the result was considered as indeterminate (i.e., in the grey zone), and these samples were deemed inconclusive. The test performances were computed using a dichotomous approach and compared regarding sensitivity, specificity, and accuracy [20]. Confidence interval was set to 95% and *p* < 0.05 was considered as statistically significant. A study workflow (Fig. 2) is provided according to the STARD guidelines [21]. Digital map (Fig. 1) was acquired from the Brazilian Institute of Geography and Statistics (IBGE) cartographic database in shapefile (.shp), which were formatted and analyzed using TerraView version 4.2, public-access software provided by the National Institute for Space Research from Brazil (www.dpi.inpe.br/terraview).

## Results

### Diagnostic performance

**Fig. 3:**
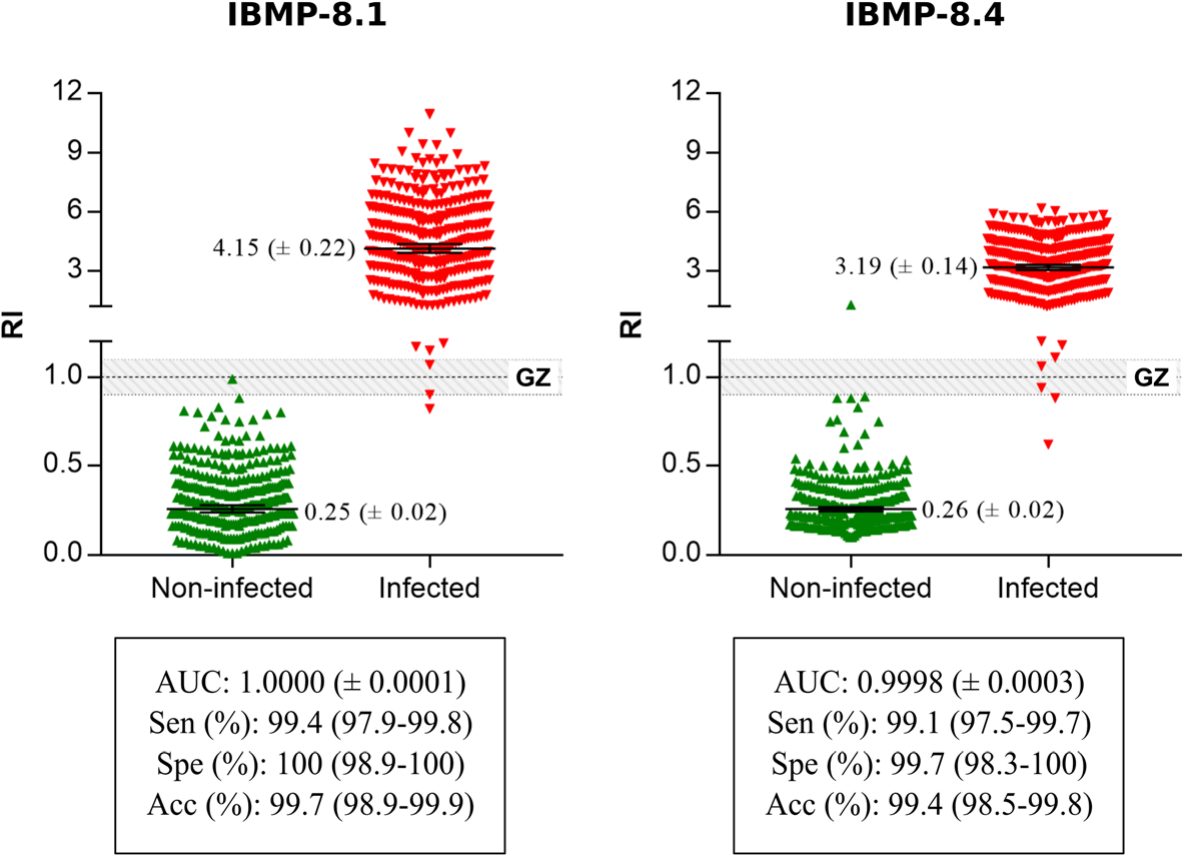
Reactivity index (RI) was obtained with serum from non-infected and *T. cruzi*-infected immigrants living in Barcelona-Spain. The cut-off value is RI = 1.0 and the grey zone is RI = 1.0 ± 0.10. Lines and whiskers represent geometric means (± 95% CI). AUC, area under curve; GZ, grey zone; Sen, sensitivity; Spe, specificity; Acc; accuracy

The reactivity index and assay performance parameters found for the IBMP-8.1 and IBMP-8.4 chimeric antigens are illustrated in Fig. 3. Based on 678 samples from non-infected and *T. cruzi*-infected individuals, the AUC (area under the curve) values were 1.000 and 0.9998 for IBMP-8.1 and IBMP-8.4 proteins, respectively, demonstrating excellent overall diagnostic accuracy. The IBMP-8.1 chimera yielded the highest diagnostic accuracy among *T. cruzi*-infected individuals, and no significant difference was observed between chimeric antigens in non-infected individuals. Sensitivity was 99.4% for IBMP-8.1 and 99.1% for IBMP-8.4. Only one positive sample was simultaneously false negative when assayed by IBMP-8.1 and IBMP-8.4 chimeric antigens. Specificity of IBMP-8.1 achieved 100 and 99.7% for IBMP-8.4. Both proteins showed an accuracy of nearly 100%. No statistically significant differences in sensitivity or specificity scores were found between the chimeric antigens. Using RI values of 1.0 ± 0.10 as the grey zone inconclusive interval, we observed two positive and one negative sample fell in the inconclusive space using IBMP-8.1 while two positive samples to IBMP-8.4 presented similar behavior. Overall, the number of inconclusive results was 0.44% for IBMP-8.1 and 0.29% for IBMP-8.4 (Fig. 3). No sample fell concomitantly inside the grey zone for IBMP-8.1 and IBMP8.4 chimeric antigens.

### Antigenic cross-reactivity

**Fig. 4:**
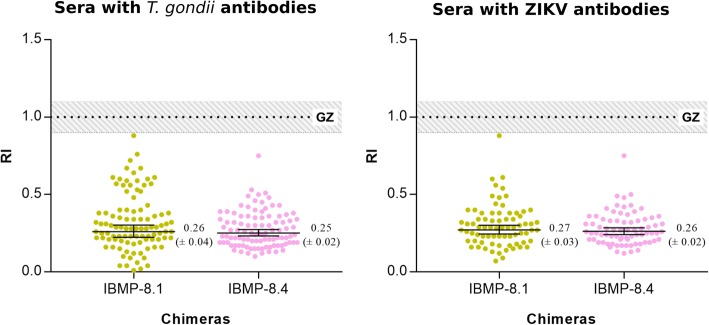
Analysis of the cross-reactivity of the IBMP-8.1 and IBMP-8.4 chimeras to sera with *Toxoplasma gondii* antibodies (*n* = 98) and Zika virus antibodies (*n* = 75). The cut-off value is 1.0 and the grey zone is RI = 1.0 ± 0.10. Lines and whiskers represent geometric means (± 95% CI). GZ, grey zone; RI, reactivity index; ZIKV, Zika virus

The RI values for sampled with *T. gondii* and ZIKV antibodies were identical to that found for non-infected individuals, indicating extremely low reactivity. No false-positive or inconclusive samples were found with IBMP chimeric antigens (Fig. 4).

## Discussion

Diagnosis of chronic CD is not a simple task due to the high genetic diversity of *T. cruzi*, which might lead to misdiagnosis [22]. In fact, *T. cruzi* parasite has remarkable genetic heterogeneity, it is classified into seven evolutionary genetic groups or discrete typing units (DTUs) termed TcI-TcVI and TcBat, with sub-classifications for regional strains [22–24]. Regional differences in sensitivity of serological tests had been reported, leading to negative CD diagnosis, mostly in non-endemic countries that receive immigrants from several endemic areas [25–28]. Therefore, a serological test should be able to diagnose CD regardless of the *T. cruzi* antigenic heterogeneity. The main benefit in the use of chimeric antigens as antigenic matrix is the increased repertoire of epitopes in comparison to non-chimeric recombinant antigens, reducing the number of false negative results. In previous studies, our group analyzed the IBMP performance to diagnose *T. cruzi*-infected individuals in several endemic and non-endemic geographical areas from Brazil [14, 15], a country where TcII is predominant [10]. Here, we used two chimeric antigens to capture specific anti-*T. cruzi* antibodies in the sera of Latin American immigrants living in Barcelona/Spain, a non-endemic setting for CD. The majority of CD-positive samples were collected from Bolivian immigrants, where TcV is the most common DTU found in Bolivia [10] and predominates in Bolivian immigrants living in Barcelona [29]. Based on the result from previous studies, we suggest that IBMP-8.1 and IBMP-8.4 antigens are able to diagnose chronic Chagas disease in areas with predominance of TcII and TcV genetic groups.

The assays exhibited high diagnostic accuracy values as demonstrated by AUC (nearly 100%), indicating a substantial discriminative power between negative and chronic CD-positive samples. Similar results were previously found in samples from several Brazilian settings both by ELISA (AUC > 99.7%) [14] and liquid microarray (AUC > 99.1%) [17]. Moreover, the reactivity index from IBMP-8.1 and IBMP-8.4 chimeric antigens, achieved from immigrants living in a non-endemic setting with CD, was higher than those previously obtained for Brazilian samples [14]. It is interesting to note that no differences were observed concerning negative samples. Further studies need to evaluate the performance of the chimeric antigens in settings where other DTUs are predominant, i.e., Argentina, Mexico and Costa Rica.

The present study showed high sensitivity and specificity for both IBMP-8.1 and IBMP-8.4, similar to those found previously using Brazilian samples [14, 15]. Also, the chimeric antigens were found to be nearly 100% accurate, suggesting that the number of misdiagnoses was negligible. In fact, only two and three CD-positive samples were misclassified when assayed with IBMP-8.1 and IBMP-8.4, respectively. In previous studies we evaluated the increase of sensitivity values and the RI signal using a multiplex methodology [17] or the equimolar mixture of the antigens [15], however no gains of were achieved. Hence, we believe that the lack of reactivity from these samples could be due to host immunological reasons or low levels of antibodies on the sera. In CD-negative samples, only one sample was classified as false-positive by IBMP-8.4 antigen. Although this sample presented low signal to IBMP-8.4 (1.28), it was classified as negative when assayed by commercial tests (RI < 0.25) and by the IBMP-8.1 chimera (0.22).

No cross-reactivity of IBMP chimeric antigens against antibodies of *T. gondii* and ZIKV was observed. Indeed, previous studies have shown extremely low reactivity of IBMP chimeric antigens for several infectious diseases, even for *Leishmania* spp. [15, 17], a pathogen phylogenetically similar to *T. cruzi*. *T. gondii* and ZIKV positive samples were used in this study due to serum bank availability and because these infectious diseases did not assay before using IBMP proteins.

## Conclusion

Our results showed a remarkable performance of IBMP-8.1 and IBMP-8.4 chimeric antigens by ELISA and suggest both antigens could also be used for CD diagnosis in immigrants living in non-endemic settings. The high accuracy of IBMP-8.1 and IBMP-8.4 chimeric antigens suggests that they are useful for CD diagnosis in individuals infected with other DTUs.

## Abbreviations

Acc: Accuracy
AUC: Area under the curve
CD: Chagas disease
DTU: Discrete typing unit
GZ: Grey zone
HRP: Horseradish peroxidase
IBMP: Molecular Biology Institute of Paraná (IBMP in Portuguese acronym)
OD: Optical density
PBS: Phosphate-buffered saline
PBS-T: Phosphate-buffered saline-0.5% Tween 20
RI: Reactivity index
ROC: Receiving operator curve
Sen: Sensitivity
Spe: Specificity
STARD: Standards for reporting of diagnostic accuracy studies
*T. cruzi*: *Trypanosoma cruzi*
*T. gondii*: *Toxoplasma gondii*
TBM: Tetramethyl-benzidine
ZIKV: Zika virus
